# Rates of Induction of Labor at 39 Weeks and Cesarean Delivery Following Publication of the ARRIVE Trial

**DOI:** 10.1001/jamanetworkopen.2023.28274

**Published:** 2023-08-10

**Authors:** Rachel Wood, Taylor Freret, Mark Clapp, Sarah Little

**Affiliations:** 1 Division of Maternal Fetal Medicine, Department of Obstetrics and Gynecology, Brigham and Women’s Hospital, Boston, Massachusetts; 2 Division of Maternal Fetal Medicine, Department of Obstetrics and Gynecology, Massachusetts General Hospital, Boston, Massachusetts

## Abstract

This cross-sectional study analyzes the rates of induction and cesarean delivery before and after the publication of A Randomized Trial of Induction vs Expectant Management (ARRIVE).

## Introduction

In August 2018, A Randomized Trial of Induction vs Expectant Management (ARRIVE) trial was published in the *New England Journal of Medicine*.^1^ This trial found that low-risk, nulliparous patients who were induced at 39 weeks’ gestation had a 16% relative risk reduction in cesarean delivery (CD) as compared with expectant management. Our objective was to evaluate if the publication of this trial was associated with observable obstetric practice changes in the US. We hypothesized the rate of 39-week induction would increase and CD would decrease for low-risk nulliparous patients after publication and dissemination of the ARRIVE trial.

## Methods

This cross-sectional study followed the Strengthening the Reporting of Observational Studies in Epidemiology (STROBE) reporting guideline. The Mass General Brigham institutional review board deemed the study exempt and informed consent was waived because the data were collected at the population level and publicly available.

We conducted a population-level interrupted time series (ITS) analysis^2^ of low-risk nulliparous patients who delivered at 39 weeks of gestation or longer between January 2016 and March 2020 using publicly available US natality data.^3^ Patients who were considered to be low risk were defined as having a singleton, vertex-presenting, nonanomalous live birth without chronic hypertension or diabetes. Demographic data were also collected, including race and ethnicity by self-report because the racial and ethnic proportions of the US population have changed over time and minoritized racial and ethnic groups experience disparities in CD rates. Self-reported race categories of Asian, Black, Native American, Native Hawaiian or Pacific Islander, more than 1 race, and White, and ethnicity categories of Hispanic or non-Hispanic were used as defined in the US Natality data.

The pre-ARRIVE period took place from January 2016 until July 2018. The primary exposure was ARRIVE trial publication in August 2018, and a 3-month dissemination period was chosen a priori. The post-ARRIVE period took place from November 2018 until March 2020, ending at this time to avoid potential confounding from the COVID-19 pandemic. The outcomes of interest were rate of 39-week induction and CD among low-risk nulliparous patients at 39 weeks of gestation or longer. Our impact model allowed for both immediate and ongoing changes in temporal trends. The primary ITS model was constructed using Poisson regression and adjusted for seasonality. We additionally controlled for the proportion of individuals 35 years or older and with body mass index (BMI; calculated as weight in kilograms divided by height in meters squared) of 30 or more, patient-level factors known to be associated with CD and changing over time.^4,5^ Checks for model robustness, including analysis excluding patients with hypertensive disorders of pregnancy, are described in the eMethods in Supplement 1. Analyses were performed using Stata version 17 (StataCorp), tests were 2-sided, and statistical significance was set at *P* < .05.

## Results

A total of 2 860 942 births were included (1 889 599 births [66%] from the pre-ARRIVE period and 971 343 births [34%] from the post-ARRIVE period). In the pre-ARRIVE period, 146 164 patients (7.7%) were aged 35 years or older, 367 068 (19.9%) had a BMI of 30 or higher, 1 419 494 patients (75.1%) self-reported their race as White, and 408 106 (21.8%) self-reported as Hispanic ethnicity. In the post-ARRIVE period, 81 890 patients (8.4%) were 35 years or older, 205 026 (21.5%) had a BMI of 30 or higher, 728 337 (75.0%) self-reported their race as White, and 221 007 (23.0%) self-reported as Hispanic ethnicity (Table). There was an immediate increase in 39-week induction rate after the dissemination period; with a 39-week induction rate of 15.0% vs an expected 13.8% based on pre-ARRIVE trends (adjusted incident rate ratio [aIRR], 1.10; 95% CI, 1.08-1.13; *P* < .001) and CD rates were significantly lower than expected (24.7% vs 25.1%; aIRR, 0.988; 95% CI, 0.978-0.997; *P* = .01) (Figure). Significant ongoing temporal changes were also noted—an increase of 0.009 (95% CI, 0.007-0.011) 39-week inductions per month (*P* < .001) and a decrease of 0.0014 (95% CI, 0.0008-0.0022) CDs per month (*P* < .001) (Figure). Induction results were robust to all diagnostic checks while CD results were robust to only some of these checks (eMethods in Supplement 1).

**Table.** Population Characteristics Before and After the Pre- and Post-ARRIVE Trial Publication

Characteristic	Patient, No. (%)	
	Pre-ARRIVE Trial (n = 1 889 599)^a^	Post-ARRIVE Trial (n = 971 343)^b^
Maternal age, y		
≤35	1 743 435 (92.3)	889 453 (91.6)
>35	146 164 (7.7)	81 890 (8.4)
BMI		
>30	367 068 (19.9)	205 026 (21.5)
>40	58 405 (3.2)	32 824 (3.4)
Race		
Asian	147 808 (7.8)	74 144 (7.6)
Black	252 103 (13.3)	130 234 (13.4)
Native American	14 004 (0.7)	7688 (0.8)
Native Hawaiian or Pacific Islander	4466 (0.2)	2782 (0.3)
More than 1 race	51 724 (2.7)	28 158 (2.9)
White	1 419 494 (75.1)	728 337 (75.0)
Maternal ethnicity		
Hispanic	408 106 (21.6)	221 007 (22.8)
Non-Hispanic	1 481 493 (78.4)	750 336 (77.2)
Delivery characteristics		
39-wk labor induction	237 545 (12.6)	164 857 (17.0)
Cesarean delivery	472 262 (25.0)	237 999 (24.5)
Birth >41 wks	296 602 (15.7)	127 794 (13.2)

Abbreviations: ARRIVE, A Randomized Trial of Induction vs Expectant Management; BMI, body mass index (calculated as weight in kilograms divided by height in meters squared).

^a^ January 2016 to July 2018.

^b^ November 2018 to March 2020.

**Figure.**
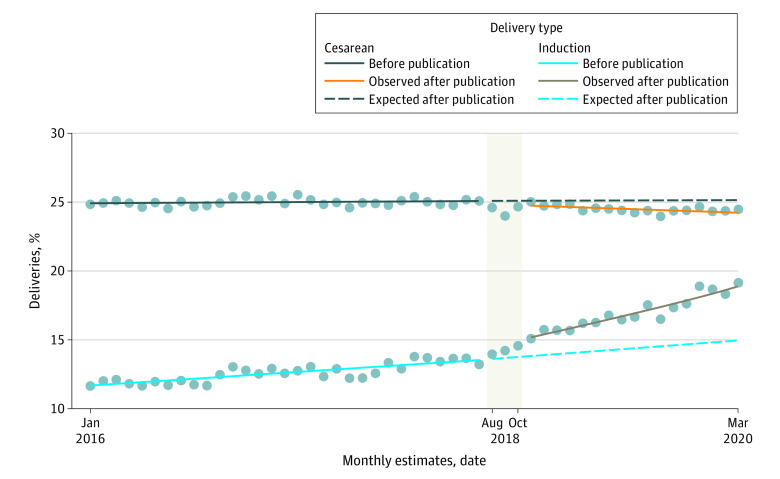
Expected and Observed Trends in 39-Week Labor Induction and Cesarean Dots indicate the monthly estimate and the shaded area is the dissemination period.

## Discussion

These findings suggest that the publication of the ARRIVE trial was associated with an increase in 39-week induction rates and a reduction in CD rates for low-risk nulliparous patients across the US. Strengths of our study include its large sample size and that it builds upon prior reports^6^ by using a robust quasi-experimental method which can overcome many limitations of traditional prestudy and poststudy design as it accounts for underlying trends already occurring in the population.

This study has limitations. Birth certificate data are limited in clinical granularity, and we were unable to replicate the exact cohort included in the ARRIVE trial. Our CD rate was slightly higher than what was seen in the ARRIVE trial, which may reflect individual-level risk factors not captured in birth certificate data. Additionally, ITS can be difficult to interpret in settings with multiple ongoing interventions that may affect outcomes, which may be why the change in CD is less robust. Future work is needed to understand whether the findings of the ARRIVE trial are being implemented similarly in different subgroups and if trends are similar in all practice settings.

## Notes

Supplement 1.
eMethods.

eReferences.

Supplement 2.
Data Sharing Statement
