# Error in the Byline

**DOI:** 10.1001/jamanetworkopen.2023.35837

**Published:** 2023-09-18

In the Research Letter titled “Rates of Induction of Labor at 39 Weeks and Cesarean Delivery Following Publication of the ARRIVE Trial,”^1^ published August 10, 2023, the middle initial was missing in Dr Freret’s name. This article has been corrected.^1^
