# Supplementary material for: Rates of Induction of Labor at 39 Weeks and Cesarean Delivery Following Publication of the ARRIVE Trial
Source: JAMA Netw Open. 2023 Aug 10;6(8):e2328274. doi: 10.1001/jamanetworkopen.2023.28274 (PMC10415960; doi:10.1001/jamanetworkopen.2023.28274)
Supplement: Supplement 1. — eMethods. eReferences. [file jamanetwopen-e2328274-s001.pdf]

Supplemental Online Content

Wood R, Freret T, Clapp M, Little S. Rates of induction of labor at 39 weeks and cesarean delivery following publication of the ARRIVE trial. *JAMA Netw Open*. 2023;6(8):e2328274. doi:10.1001/jamanetworkopen.2023.28274

eMethods.

eReferences.

This supplemental material has been provided by the authors to give readers additional information about their work.

© 2023 Wood R et al. *JAMA Network Open.*

eMethods

An interrupted time series analysis (ITS) was conducted to determine the association between the publication of the ARRIVE trial and 39-week induction of labor and cesarean delivery (CD) rates in low-risk nulliparous patients in the United States[1].

The cohort included patients delivering non-anomalous, liveborn, singleton, vertex infants between January 2016-March 2020 at ≥ 39 weeks gestation, with no reported maternal diabetes or chronic hypertension. Births were included only if information was available regarding mode of delivery (cesarean versus vaginal), gestational age at delivery, and induction of labor.

Poisson models were constructed using the following equation to generate relative changes in rates for the variables of interest[2]:

$Yt = \beta 0 + \beta 1\ T + \beta 2Xt + \beta 3TXt + B4Xp + \varepsilon$

where:

– Yt: outcome at time t

– T: time in months

– Xt: indicator variable for the pre- or post-dissemination period

– Xp: patient-specific factors, adjusted model only

– ε: error term, estimated from bootstrapping (n=50 iterations)

$\beta 0$ represents the baseline level at T = 0

© 2023 Wood R et al. *JAMA Network Open.*

$\beta 1$ is interpreted as the change in outcome associated with a time unit increase (representing the underlying pre-intervention trend)

$\beta 2$ represents the immediate effect of the intervention on the outcome. Incidence rate ratios reported are for the $\beta 2$ coefficient.

$\beta 3$ indicates the slope change following the intervention.

An adjusted model was then constructed controlling for patient level factors, maternal age ≥35 years old (categorized as <35 years old and ≥35 years old) and obesity (categorized as BMI <30 or ≥30) as these factors are known to be increasing in the United States population and independently associated with CD.

The interrupted time series model was constructed using the following time periods:

- Pre-period: January 2016-July 2018 (31 months), spanning from the month expanded birth certificate data is first available online for all US States until the dissemination period
- Trial dissemination period: 3 months (August-October 2018) including the study's first publication online at NEJM.org and American College of Obstetricians and Gynecologists' (ACOG) statement published online supporting 39-week induction for low risk nulliparas as a reasonable choice for clinicians to offer patients[1,3]
- Post-period: November 2018-March 2020 (17 months), month immediately following dissemination period until March 2020, the onset of the Covid-19 pandemic in the United States when practice patterns may have changed for reasons unrelated to this study[4].

<u>Checks for Robustness</u>

© 2023 Wood R et al. *JAMA Network Open.*

1. **Analysis excluding patients with hypertensive disorders of pregnancy:** In the ARRIVE trial, patients were enrolled in the 38th week of pregnancy and excluded if already carried a diagnosis of gestational hypertension, pre-eclampsia, or eclampsia (hypertensive disorders of pregnancy); however, development of hypertensive disorders of pregnancy was one of the secondary outcomes of interest and thus patients remained in the trial if they developed these diagnoses after randomization[1]. CDC Natality birth certificate data does not give any temporal information regarding when the diagnosis of hypertensive disorders of pregnancy were made and thus these patients were included in the primary analysis and excluded in a planned secondary analysis with no significant changes noted in results 39-week induction of labor or cesarean delivery rates when these patients were excluded.

2. **Adjustment of impact model:** The primary model was constructed to allow a one-time level change as well as a change in slope of the outcome of interest. A secondary model was also constructed allowing only for a one-time level change in the month immediately following the dissemination period with no significant change in results.

3. **Placebo tests**: In which artificial dissemination periods and subsequent pre-period and post-periods were constructed prior to the true dissemination period. We started with placebo dissemination period of months 4,5,6 (April, May, June 2016) we then advanced the model by one month for two years, avoiding any overlap with the true dissemination period, which resulted in in a total of 24 placebo tests. Point estimates were compared among the placebo tests and the main analysis estimate to evaluate whether the observed association in the true interrupted time series analysis was significantly different than one measured at a time chosen at random. Fisher's Exact test was used to determine the significance of the observed main

© 2023 Wood R et al. *JAMA Network Open.*

effect in relation to the placebo tests. The change in 39-week induction during the study dissemination period was significantly greater than the placebo dissemination periods; however, the change in cesarean delivery during the study dissemination period was of a similar magnitude to changes seen in several of the placebo time periods.

© 2023 Wood R et al. *JAMA Network Open.*

eReferences

1. Grobman WA, Rice MM, Reddy UM, et al. Labor Induction versus Expectant Management in Low-Risk Nulliparous Women. *New England Journal of Medicine*. 2018;379(6):513-523.
2. Bernal JL, Cummins S, Gasparrini A. Interrupted time series regression for the evaluation of public health interventions: a tutorial. Int J Epidemiol. 2017 Feb 1;46(1):348-355. doi: 10.1093/ije/dyw098. Erratum in: Int J Epidemiol. 2020 Aug 1;49(4):1414. PMID: 27283160; PMCID: PMC5407170.
3. Committee on Obstetric Practice. American College of Obstetricians and Gynecologists: Clinical Guidance for Integration of the Findings of The ARRIVE Trial: Labor Induction Versus Expectant Management in Low-Risk Nulliparous Women. Published August, 2018, re-affirmed October 2022.
4. Chmielewska B, Barratt I, Townsend R, et al. Effects of the COVID-19 pandemic on maternal and perinatal outcomes: a systematic review and meta-analysis. *Lancet Global Health*. 2021; 9: e759–72.

© 2023 Wood R et al. *JAMA Network Open.*
