# Supplementary material for: Rates of Induction of Labor at 39 Weeks and Cesarean Delivery Following Publication of the ARRIVE Trial
Source: JAMA Netw Open. 2023 Aug 10;6(8):e2328274. doi: 10.1001/jamanetworkopen.2023.28274 (PMC10415960; doi:10.1001/jamanetworkopen.2023.28274)
Supplement: Supplement 2. — Data Sharing Statement [file jamanetwopen-e2328274-s002.pdf]

Data Sharing Statement

Wood. Rates of Induction of Labor at 39 Weeks and Cesarean Delivery Following Publication of the ARRIVE Trial. *JAMA Netw Open*. Published August 10, 2023.
doi:10.1001/jamanetworkopen.2023.28274

Data
Data available: No

Additional Information
Explanation for why data not available: This data is already publicly available at https://wonder.cdc.gov/natality.html
